# Cellular effects induced by 17-β-estradiol to reduce the survival of renal cell carcinoma cells

**DOI:** 10.1186/s12929-016-0282-z

**Published:** 2016-09-29

**Authors:** Sheng-Tang Wu, Wei-Chi Ku, Chi-Jung Huang, Yen-Chieh Wang, Chih-Ming Lin, Shao-Kuan Chen

**Affiliations:** 1 Division of Urology, Department of Surgery, Tri-Service General Hospital, National Defense Medical Center, Taipei, Taiwan; 2 School of Medicine, College of Medicine, Fu Jen Catholic University, New Taipei, Taiwan; 3 Department of Biochemistry, National Defense Medical Center, Taipei, Taiwan; 4 Department of Medical Research, Cathay General Hospital, Taipei, Taiwan; 5 Department of Surgery, Sijhih Cathay General Hospital, No. 2, Ln. 59, Jiancheng Rd., Sijhih Dist., New Taipei, 22174 Taiwan; 6 Department of Surgery, Cathay General Hospital, Taipei, Taiwan

**Keywords:** Renal cell carcinoma, 17-β-estradiol, Estrogen receptors, Oxidative stress, DNA damage

## Abstract

**Background:**

Renal cell carcinoma (RCC) is an adult malignancy with 2:1 men-to-women ratio, which implies the possible role of sex hormones in RCC carcinogenesis. One of the predominant sex hormones in women before menopause, 17-β-estradiol (or E2), may regulate RCC growth by cellular mechanisms that are still not fully understood.

**Methods:**

The expression levels of E2 receptors (ER1 and ER2) were determined in different RCC cell lines. The DNA damage response induced by E2 was determined by a DNA double-strand break marker γH2AX. To study the possible effect of E2 on oxidative stress response, RCC cells were stained with 2,7-dichlorofluorescein diacetate and analyzed by flow cytometry. Upregulation of nuclear factor (erythroid-derived 2)-like 2 (Nrf2) ser40 phosphorylation in response to oxidative stress was detected by immunoblotting. Finally, annexin V/propidium iodide (PI) double staining assay was used to determine E2-induced cellular apoptosis.

**Results:**

Variable expression of ER1 and ER2 were found in the RCC cell lines studied (786-O, A498, and ACHN), in which ACHN and A498 showed highest and lowest ER expression, respectively. In A498 cells, E2 induced DNA double-strand breaks with positive staining of γH2AX. On the other hand, the level of reactive oxidative species were elevated in ACHN cells after E2 treatment. The E2-induced oxidative stress also induced the Ser40 phosphorylation and nuclear translocation of Nrf2. Finally, we also demonstrated that E2 induced apoptosis as revealed by annexin V/PI double staining.

**Conclusions:**

In this study, we demonstrated the cellular effects of E2 on DNA repair, ROS production as well as Nrf2 activation, and apoptosis in RCC cell lines. Together these cellular alterations may contribute to the reduced viability of RCC cells following E2 treatment.

## Background

Renal cell carcinoma (RCC) is the most common solid neoplasm of the kidney in adult human. This kidney cancer exhibits a 2:1 male-to-female ratio with a better survival rate in younger women [16]. Thus, there appear to be hormonal effects on the development, progression, and treatment of RCC [31, 33].

In RCC, altered chemosensitivity mediated by 17-β-estradiol (E2) has been reported, so adjuvant therapy with E2 is possible as in other human diseases [24, 45]. The molecular mechanisms of the action of E2 in RCC cells have been reported in recent years [8, 10, 13, 43, 45]. However, diverse genetic alterations are linked to renal cancer progression and to our ability to predict its risk [13, 23]. Targeted therapies incorporating inhibitors of the vascular endothelial growth factor (VEGF) pathway is the major treatment for patients with metastatic RCC [18, 20, 49]. For example, anti-angiogenic drugs such as sunitinib can nullify alterations in the microvascular properties of some human cancers [35]. This inhibition reduces tumor vascularization, and leads to tumor shrinkage. Clinically, cellular adaptations to VEGF-targeted anti-angiogenic therapy might induce drug resistance in RCC after transient disease stabilization, which limits the therapeutic efficacy of this approach and has been the major problem for this treatment. Therefore, a rational and efficacious treatment regimen for treating patients with RCC is needed [4, 14].

E2, is known to exert a protective effect beyond its classical endocrine role in several malignant diseases including neurodegenerative disorders, esophageal cancers, colorectal cancers, lung injuries, and coronary artery diseases [11, 40, 42, 46]. Many cellular functions can be improved by treatment with E2 alone or in combination with its receptors [3, 25]. For example, E2 can induce an increase in sensitivity to oxidative DNA damage through an ER-dependent pathway [28]. Moreover, the oxidative stress has been correlated to the phosphorylation of nuclear factor (erythroid-derived 2)-like 2 (Nrf2) at Ser40 [5, 29]. Therefore, the aim of this study was to understand whether E2 might induce different cellular effects on RCC cells. Briefly, changes in DNA repair mechanisms, oxidative stress response, and viability were determined to evaluate the altered cellular effects.

## Methods

### Cell culture and genetic characteristics of RCC cell lines

The incubation condition and clinical status of three RCC cell lines (ACHN, 786-O, and A498) were based on information disclosed on the website (https://www.atcc.org) of the American Type Culture Collection (Manassas, VA, USA). Their genetic characteristics are known from previous reports [9, 47]. In experiments that involved treatment with a specific chemical agent (or target hormone), subconfluent cell cultures were incubated with the indicated concentrations of E2 (E8875, Merck, Darmstadt, Germany) for the indicated times as our previous report [8]. Control cells were treated only with the solvent dimethyl sulfoxide (DMSO).

### Immunodetection of estrogen receptors and Nrf2 from RCC cells

The protein levels of two estrogen receptors (ERs), ER1 and ER2, in RCC cells were determined by western blot analysis. For this, protein extracts of each RCC cell line were prepared with Pro-prep Protein Extraction Solution (iNtRON Biotechnology, Seongnam, South Korea) according to the manufacturer’s protocol with minor modifications. Briefly, the cell lysate was centrifuged for 10 min at 12,000 g to remove cellular debris. When analyzing the different protein fractions from the cytoplasm and nuclei, the different cellular compartments were extracted and separated using NE-PER Nuclear and Cytoplasmic Extraction Reagents (Thermo Fisher Scientific, Rockford, IL, USA) according to the manufacturer’s instructions. Each protein concentration was determined using a BCA Protein Assay (Thermo Fisher Scientific). When performing western blotting, protein lysates were separated by sodium dodecyl sulfate polyacrylamide gel electrophoresis and transferred to polyvinylidene difluoride (PVDF) membranes. Then, each PVDF membrane was incubated with primary antibodies (anti-ER1, ab37438 and anti-ER2, ab3576; both from Abcam, Cambridge, MA, USA) for 1 h and then for another 1 h with horseradish peroxidase-labeled secondary antibody (L3012 for goat anti-rabbit IgG; Signalway Antibody, College Park, MD, USA) at room temperature. Target signals were exposed and enhanced using Western Lightning Plus-ECL Enhanced Luminol reagent (PK-NEL105, PerkinElmer, Waltham, MA, USA). In addition, the total Nrf2 and its phosphorylated form at Ser40 (phospho S40) in different cellular compartments were determined using specific antibody (ab62352 and ab76026, respectively; Abcam) from 10 μg aliquots of individual proteins. Target signals were amplified using the VECTASTAIN ABC-AmP kit (AK-6602, Vector Laboratories, Burlingame, CA, USA). The level of glyceraldehyde 3-phosphate dehydrogenase (anti-GAPDH antibody; AM4300, Life Technologies, Carlsbad, CA, USA) was determined as a loading control for whole-cell lysates. An anti-α-tubulin antibody (sc-5286, Santa Cruz Biochemicals, Dallas, TX, USA) was used as an internal control for cytoplasmic protein levels, and lamin A/C (anti-lamin A/C antibody; sc-7292, Santa Cruz Biochemicals) was used as an internal control for nuclear protein levels. Images of immunoblots were captured using a FluorChem FC2 system (Alpha Innotech, Santa Clara, CA, USA). Cell lysates of two breast cancer cell lines (T47-D and MCF-7) gifted from Prof. Shih-Ming Huang (Department and Graduate Institute of Biochemistry, National Defense Medical Center, Taiwan) served as positive controls for ER1 and ER2.

### Assay for the formation of phosphorylated histone H2AX (γ-H2AX) foci

After incubation for 24 h of 5 × 10^4^ cells per well, DNA double strand breaks (DSBs) were induced in A498 cells using etoposide as described in our previous report [17] and observed in the presence or absence of E2. The ability to repair DSBs was determined by staining with an anti-γ-H2AX antibody using an OxiSelect DNA Double Strand Break Staining Kit (Cell Biolabs, San Diego, CA, USA) according to the manufacturer’s instructions with some minor changes. Briefly, cells were allowed to recover by removing the DSB inducer for the indicated time; the exposed DSBs (shown as γ-H2AX foci) appeared as green fluorescence and nuclei were counterstained with 4′,6-diamidino-2-phenylindole. These fluorescent images were all detected using an Olympus IX70 fluorescence microscope (Olympus, Tokyo, Japan).

### Detection of intracellular oxidative stress by flow cytometry

Intracellular oxidative stress induced by E2 treatment was determined by flow cytometry using 2′,7′-dichlorfluorescein-diacetate (DCFH-DA) as a sensitive nonfluorescent precursor dye according to a published standard procedure [1]. Cells were seeded on 6-well plates at 1.25 × 10^5^ cells per well and cultured overnight as our previous report [8]. Then, cells were treated with E2 (7 or 28 μM) for 24 h and incubated with 50 μM DCFH-DA for 20 min in an incubator following an appropriate wash with phosphate-buffered saline (PBS). Cells were treated without E2 (DMSO alone) as a negative control or with 1 mM H_2_O_2_ for 30 min as a positive control. Cells were then washed again with PBS, incubated with 0.25 % trypsin- ethylenediaminetetraacetic acid for 1 min, and then quenched with minimum essential medium with 10 % fetal bovine serum to stop trypsinization. Detached cells were collected by centrifuging at 500 *g* for 5 min and resuspended in PBS. The fluorescence was determined by Flowcytometer FACSCalibur (BD Biosciences, Franklin Lakes, NJ, USA), with excitation at 480 nm and emission at 525 nm.

### Detection of apoptosis

To identify apoptosis-positive cells, cells were stained with an Annexin V/propidium iodide (PI) double staining assay, with a fluorescein isothiocyanate (FITC) Annexin V Apoptosis Detection Kit I (BD Biosciences), according to the manufacturer’s protocol. Cells with various treatments were washed with buffer (20 mM Tris pH 7.4, 150 mM NaCl, 1 mM CaCl_2_) after incubation for 15 min in binding buffer containing 5 μL of Annexin V–FITC and 5 μL of PI at room temperature in the dark. Finally, fluorescent images of Annexin V–FITC binding and PI incorporation were detected using the previous fluorescence microscope.

## Results

### Diverse expression levels of ERs in different RCC cell lines

**Fig. 1:**
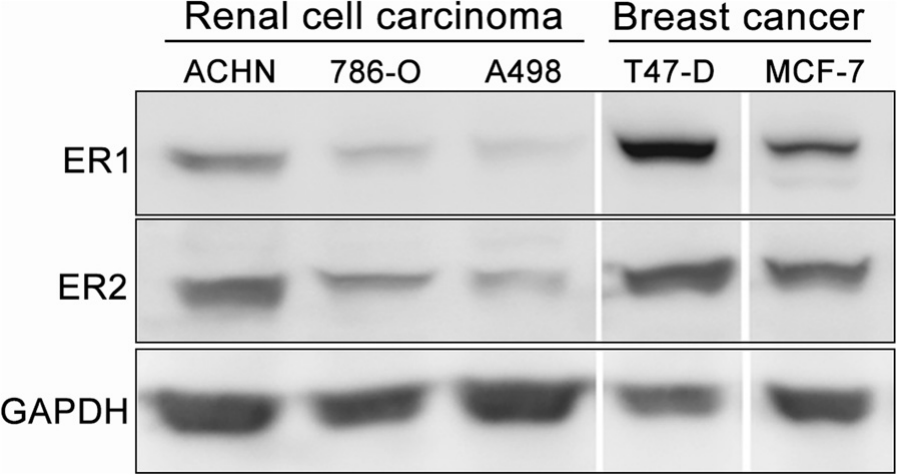
Immunodetection of estrogen receptors in cell lines of renal cell carcinoma by Western blots. Protein lysates were prepared from cell lines of renal cell carcinoma (ACHN, 786-O, and A498) and breast cancer (T47-D and MCF-7), which served as positive controls. GAPDH (glyceraldehyde-3-phosphate dehydrogenase) was the internal control. *ER1* estrogen receptor 1, *ER2* estrogen receptor 2

Three RCC cell lines (ACHN, 786-O, and A498) were cultured to detect the expression levels of ER1 and ER2 by western blot analysis (Fig. 1). Among these three cell lines, ACHN cells expressed the highest protein levels of ERs and A498 had the lowest expression, as reported previously for mRNA levels [8]. Therefore, these two RCC cell lines were subsequently analyzed for following cellular effects.

### Induction of DNA repair by E2 in A498 cells

**Fig. 2:**
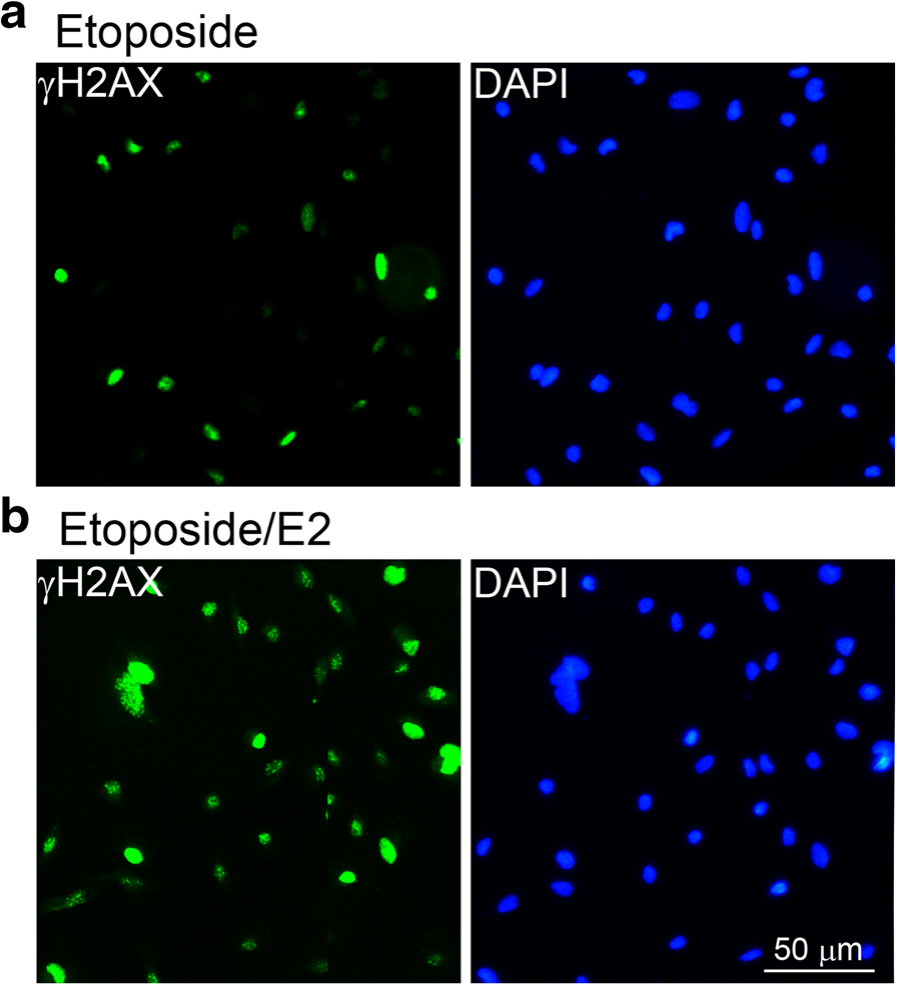
DNA repairing ability of A498 cells with treatments of etoposide and E2. A498 cells treated respectively with **a** etoposide (50 μM) alone or **b** etoposide (50 μM) and E2 (70 nM). *Green*, γH2AX-positive cells; *blue*, DAPI (4′,6-diamidino-2-phenylindole. The *scale bar* represented the 50 μm. E2, 17-β-estradiol

A498 cells (low expressions of ERs, wild-type p53, and VHL-null) had different properties of DNA repair under E2 treatment. As shown in Fig. 2, over 90 % (37 of 40) cells with active DNA repair were observed with green fluorescence for γH2AX under E2 treatment; whereas, less percentage of cells (44 %, 18 of 41) showed fluorescence-positive in the etoposide-treated group. This indicated that E2 could improve DNA repair, even though this RCC cell line had low expression levels of the ERs.

### Induction of oxidative stress by E2 in ACHN cells

**Fig. 3:**
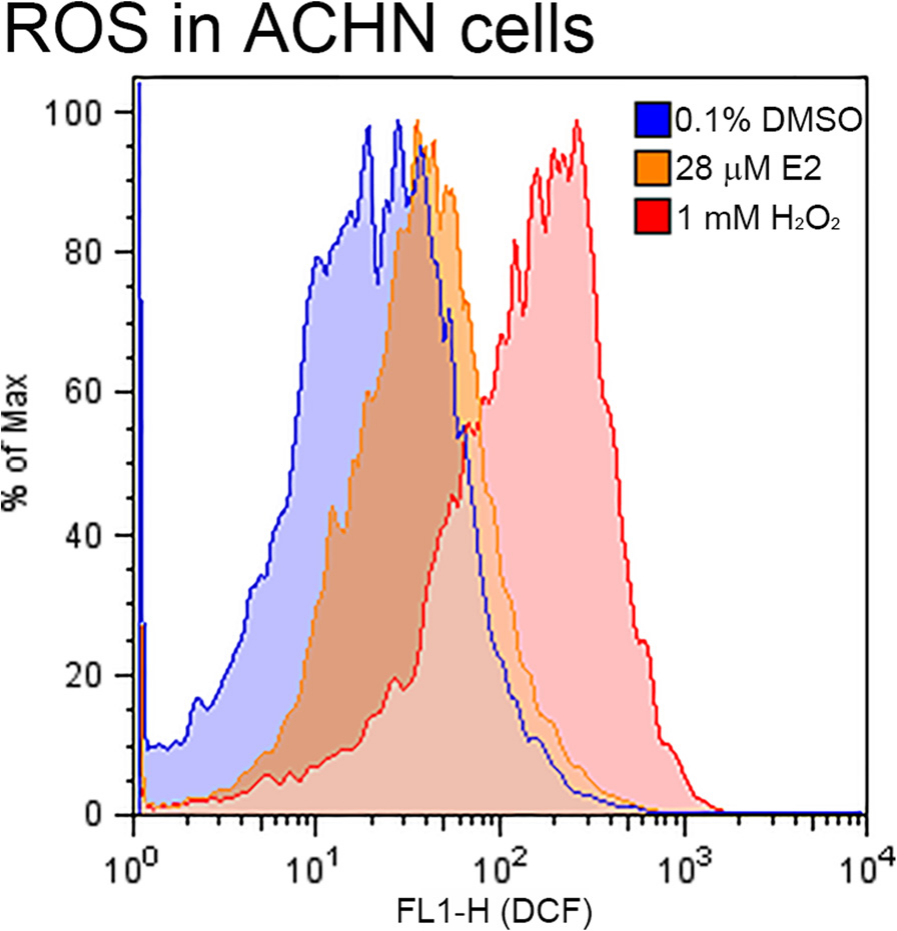
Induction of oxidative stress by E2. Reactive oxidative species (ROS) formation in ACHN cells were detected by flow cytometry (28 μM E2, *orange line*). DCFH-DA-pretreated cells as ROS-positive were incubated with 50 μM DCFH-DA for 30 min at 37 °C. *Blue line*, 0.1 % DMSO (negative control); *red line*, 1 mM H_2_O_2_ (positive control). E2, 17-β-estradiol

**Fig. 4:**
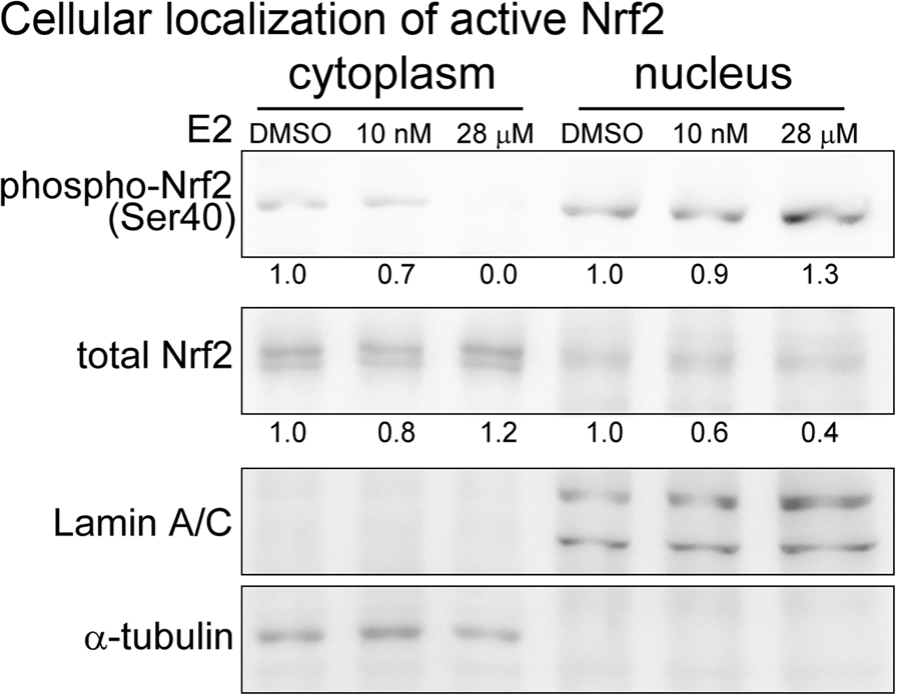
Phosphorylation of nuclear Nrf2 by E2. Cytoplasm and nuclear extracts were respectively isolated from ACHN cells. Each compartment was immunodetected with antibody raised against phospho Nrf2 (Ser40), lamin A/C (for nuclear control), and α-tubulin (for cytosol control). Each protein level relative to individual control was determined by densitometry and shown on the bottom of each band. E2, 17-β-estradiol

Reactive oxygen species (ROS), known to reflect oxidative stress, can cause significant damage to cell structures [33]. E2-induced ROS levels in ACHN cells (high ERs, wild-type p53, and VHL-positive) were detected using the DCFH-DA probe. As shown in Fig. 3, ACHN cells showed an increased trend in intracellular ROS levels when cells were treated with E2 (28 μM) for 24 h compared with the DMSO-treated negative control cells. In addition, activation of the Nrf2 transcription factor was observed (Fig. 4). Briefly, increased phosphorylation of nuclear Nrf2 (1.3 folds, in comparing to the cells treated with DMSO only) were detected in ACHN cells treated with E2 (28 μM), using immunoblot analysis.

### Augmentation of apoptosis by E2 in ACHN cells

**Fig. 5:**
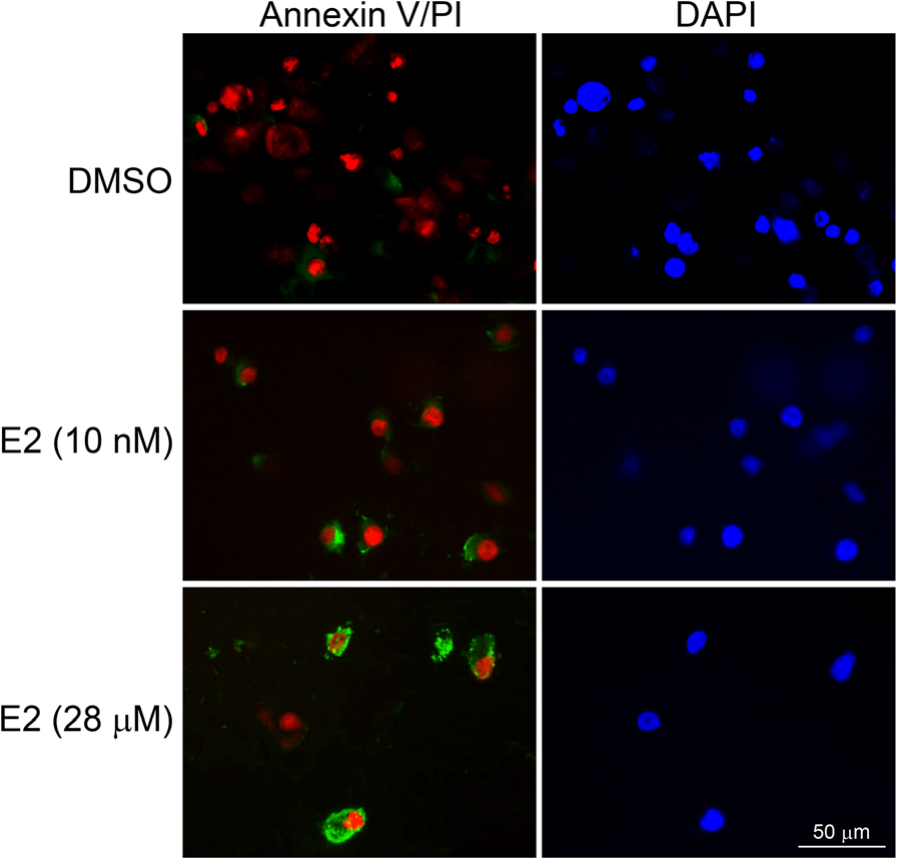
Apoptotic effect of E2 on ACHN cells. ACHN cells were treated without E2 (DMSO) and with E2 (10 nM and 28 μM). Annexin V, *green*; and PI (propidium iodide), *red*. E2, 17-β-estradiol. The *scale bar* represented the 50 μm

We next analyzed the apoptotic effects of E2 in ACHN cells by detecting Annexin V-positive cells. As shown in Fig. 5, ACHN cells treated with E2 (28 μM) could induce the higher binding rates (70 %, 7 of 10 E2 (10 nM)- and 75 %, 3 of 4 E2 (28 μM)-treated cells) of Annexin V to phosphatidyl serine at the surface of each cell. Conversely, only 10 % (2 of 20) cells shown Annexin V-positive in the DMSO-treated control group.

## Discussion

Although the number of approved treatments for patients with advanced RCC continues to grow, additional work is still needed to delineate the optimal target drug or combination of agents for each subtype of this cancer [36]. The therapeutic management of RCC is mainly guided by the cancer stage and the degree of tumor dissemination, which affect the prognosis for patients directly [7]. Regardless of the clinic availability of the novel targeted therapeutics, and the favorable outcomes, however, complete or durable responses have been only rarely noted. Nearly all treated patients even now will develop drug resistance to any targeted treatment. Approximately 70 % of patients respond to therapy initially and the remaining 30 % show primary resistance (intrinsic resistance) [6, 15]. The 70 % of patients who show initial response, durable responses are rare, and acquired resistance (extrinsic resistance) to treatment develops in almost all of them in a median of 6–15 months [6, 15, 32]. However, it remains a challenge in the effective treatment of RCC and suggests that effective therapies for RCC need to be improved [12].

Autophagy mediated by E2 might have inhibited the growth rate of ACHN cells in the present study [8]. In addition, the activities of ERs have been correlated with those of VHL and p53 in many studies [2, 19, 41]. Therefore, the genetic status of VHL and p53 expressions of RCC cells should be considered when E2 is applied to treat RCC. p53-mediated transcription pathways are involved in ER-related pathways [26]. Loss of VHL may attenuate the DNA-damage response and result in the persistence of DSBs [27]. In the present results, A498 cells expressing wild-type p53 but with no VHL expression could repair etoposide-induced DNA damage in the presence of E2. The increased numbers of γH2AX-positive A498 cells implied that lower levels of ERs would not decrease their DNA repair capacity and that E2 treatment could facilitate repair of the DNA breaks even the cells were in the absence of VHL expression. This was similar to the conclusions of other authors who reported that complexes of E2 with ER1 or ER2 modulated the expression of many genes, including those involved in DNA repair [3, 22, 25, 39]. Moreover, the decreased DSBs would be obvious in the ACHN cells with wild type VHL expression. Here, we also found that ACHN cells expressed high levels of ERs. This might indicate that ACHN cells might be sensitive to E2 treatment and that this could induce many other cellular effects. This perspective also arose in other reports showing that E2 might be correlated to the development of this cancer [40, 45].

The highly conserved mechanism of autophagy is essential when cells are under stress, including that induced by ROS [44]. We reported previously that autophagosomes were observed in ACHN cells in the presence of E2 [8]. Sobočanec et al. and Zhu et al. recently reported that E2 could mediate some antioxidant enzymes via the Nrf2–Keap1 pathway [37, 48]. It will be agreed with our data which demonstrate an increased ROS under the E2 treatment. Here, we found that E2 could induce ROS production in ACHN cells. These E2-induced ROS might activate the Nrf2 pathway to reduce the oxidative stress and induce genotoxicity [30, 38]. Subsequently, sequential cell responses, autophagy, and apoptosis changed the fate of the ACHN cells. These findings were confirmed by the expression of activated nuclear Nrf2, a key transcription factor that regulates antioxidant defenses [21]. We found increased phosphorylation of Nrf2 in the nuclei of E2-treated ACHN cells, which might cause a delay in proliferation and induce apoptosis, similar to other cancers [34]. Taken together, our results indicate that Nrf2 activation may be necessary for this E2-induced autophagy and apoptosis. Combination therapy with E2 and target therapeutic agents may be a safe and potential practice clinically to improve the tolerance and treatment duration of target therapy.

## Conclusions

Adjuvant therapy with sex hormones could be beneficial in many human diseases [8, 24]. Here, we demonstrated the cellular effects of E2 on DNA repair, ROS production as well as Nrf2 activation, and apoptosis in RCC cell lines. It implies that these cellular alterations may contribute to the reduced viability of RCC cells following E2 treatment.

## Abbreviations

DCFH-DA: 2′,7′-dichlorfluorescein-diacetate
E2: 17-β-estradiol
ER1: E2 receptor 1
ER2: E2 receptor 2
Nrf2: Nuclear factor (erythroid-derived 2)-like 2
RCC: Renal cell carcinoma
ROS: Reactive oxygen species
VEGF: Vascular endothelial growth factor
